# Performance Comparison of Public Hospitals Between 2014 and 2018 in Different Regions of Guangdong Province, China, Following 2017 Medical Service Price Reforms

**DOI:** 10.3389/fpubh.2021.701201

**Published:** 2021-06-30

**Authors:** Kai-Yuan Weng, Feng Xia, Wen-Qi Lin, Yi-Bao Wang

**Affiliations:** ^1^School of Public Management and Policy, China University of Mining and Technology, Xuzhou, China; ^2^College of Pharmacy, Guangdong Pharmaceutical University, Guangzhou, China; ^3^Medical Insurance Office, The Second Affiliated Hospital Zhejiang University School of Medicine, Hangzhou, China

**Keywords:** public hospitals, medical service prices, performance, regional differences, China

## Abstract

This study analyzed performance of public hospitals and regional differences in performance following reform of medical service prices in Guangdong province, China. From three cities in four regions, we randomly selected a total of 12 traditional Chinese medicine hospitals and 12 general tertiary hospitals. Six questionnaires were completed by the hospitals, using 2014–2018 internal data. Principal components analysis was used to compare performances of the hospitals and regions following price reform. The extent to which medical service prices were adjusted varied considerable for different procedures in the same region and for the same category of procedures among regions. After reform, compensation for medical services in public hospitals reached the target of 80%, except in the Western region. However, annual growth of costs to patients was generally above 4%; the burden on patients was not alleviated by fee control. Reforms were more effective for comprehensive than Chinese traditional medicine hospitals. Performance scores of general hospitals in the Pearl River Delta, Eastern, Western, and Northern regions were 1.24, 1.16, −0.22, and −1.01, respectively. This is consistent with ranking by level of economic development of each region. The government should implement a regional medical service pricing mechanism. Additionally, comprehensive and traditional Chinese medicine hospitals should each have appropriate pricing policies. Future policies should focus on controlling costs incurred by patients.

## Introduction

In recent years, the Chinese government has enacted significant medical service price reforms in public hospitals to solve the problem of expensive medical treatment. The price of medical services is the monetary expression of the medical service's value, including outpatient, hospitalization, examination, treatment, testing, surgery, and so on. Previous studies have shown that medical service prices are closely related to quality of life (1, 2). Prior to the medical service price reform, patients who received drugs from urban public hospitals in the Guangdong Province of China were required to pay 12–15% of the drug price. However, on July 1, 2017, public tertiary hospitals in Guangdong Province enacted medical service price reforms, by abolishing drug costs to patients. The policy stipulates that 80% of the income from drug reductions will be compensated by adjusting the hospital medical prices. In addition, the government subsidies 10%, and the hospital itself bears 10%. The policy optimizes the income structure of hospitals by reducing the proportion of drug revenue and increasing the income of technical services such as diagnosis, surgery and treatment (3).

The price adjustment policy affects a large number of medical services, and the adjustment range has changed greatly (4). This has far-reaching impact on hospitals, patients, and medical insurance funds (5). In this reform, the adjustment and formulation of local medical service prices are the responsibility of prefecture-level cities. Therefore, the prices of medical services may vary among cities and the implementation of policies may vary by region.

There exists relevant research on the implementation in other provinces of China. Zhang found that hospitals' income structure improved significantly after the price adjustment in Sichuan Province (6). A survey of Qingdao public hospitals found that hospital operating costs were more reasonable after the price reform (7). Tang and colleagues showed that the Nanjing government has a good control over price adjustments, and the compensation mechanism is reasonable (8).

Different countries have different pricing mechanisms for medical services. The price of medical services in some countries is controlled by the government, such as Canada, Germany, and Japan. In other countries, the price of medical services is negotiated by doctors, medical institutions, and insurance companies, such as in the United States. In 1989, Professor Xiao of Harvard University established a Resource-Based Relative Value Scale (RBRVS) by which to estimate the price of more than 7,000 medical service items. Subsequently, the RBRVS model was widely used by other countries (9–13). In 2006, the United States introduced multiple procedures for payment reduction (MPPR). When a patient receives multiple related procedures (e.g., CT, MRI), full price is charged for the most expensive procedure, with the other procedures being charged at a discount (14–17). In Japan, hospitals charge patients according to the Medical Insurance Points Table, with a score of 10 Yen per point (18, 19). In the UK, 97% of health care funding is obtained from taxes paid by residents to the government. Almost all medical service professionals are directly employed by the government, and residents receive free medical care at the point of service (20–22).

There are great differences between China and other countries in the price management model of medical services. Haiyin and colleagues suggested that China's medical service price reform has strong management and control capabilities, but the concept of performance-based payment, as used in the United States, is also of potential value (23).

To date, no studies have used hospital data to analyze the price of Chinese medical services. In China, most researchers have engaged in theoretical discussions. Some researchers have used a small number of indicators, such as compensation rate and balance rate, to conduct cross-sectional analysis of a hospital or region (24–26). However, these studies rarely compared differences among the regions involved. In addition, the use of statistical methods is rarely seen in the literature on the price of medical services. In the current study, the internal data of 24 hospitals in 12 cities in Guangdong Province in the past 5 years were assessed using principal components analysis to compare regional differences in reform performance, including in-depth analysis of differences in the implementation effects of policies in different regions. This study thereby addresses a current research gap regarding China's current medical service price reforms.

There are three advantages to the principal components analysis used in this study. First, statistical results permit differences among regions in the effects of reforms to be clearly elucidated. Second, performance comparisons among the same type of hospitals enables testing of the extent of each hospital's policy implementation. Third, it is possible to characterize differences between general hospitals and Chinese medicine hospitals. By this approach, we illustrate the shortcomings in the implementation of the policy, and propose improvement measures.

## Materials and Methods

### Data Source

Guangdong Province can be divided into the Pearl River Delta region (e.g., Guangzhou, Foshan, Zhongshan), the Eastern region (e.g., Shantou, Chaozhou, Jieyang), the Western region (e.g., Yunfu, Maoming, Yangjiang), and Northern regions (e.g., Meizhou, Zhaoqing, Qingyuan) based on geographic, but with the regions varying in economic development. In this study, three cities were randomly selected from each region, and within each city, municipal people's hospitals and municipal traditional Chinese medicine hospitals were selected as research objects. Therefore, 12 comprehensive tertiary hospitals and 12 traditional Chinese medicine hospital were considered in this study. These hospitals have robust administrative and information systems, and thus provided authentic and reliable data.

With the agreement of the government and the hospitals, we designed questionnaires, and asked each hospital to extract data from its internal information system and financial statements. In the process of questionnaire design, we referred to other provinces' price assessment methods and related literature (27–29). After two rounds of group discussions, six questionnaires were initially generated that addressed the following topics: hospital drug revenue, basic status of hospital medical service price compensation, hospital medical income classification, hospital workload status, patient expenses, and charges for medical procedures. After preparing the questionnaires, they were further revised and improved in consultation with six relevant experts who are well-known in the field of health management. Then, we selected three hospitals in which to conduct a preliminary survey using the prepared questionnaires. It was verified that the questionnaires could be implemented in practice and fully collected the information required for the project. The hospitals were required to provide the completed questionnaire and financial statements for 2014–2018.

### Data Analysis

There was a 100% response rate by the hospitals, but some hospitals provided incomplete data, such that only 60.5% of data were initially complete. To address missing data, we contacted the hospitals by phone and email and thereby completed the missing items. However, the data of four hospitals differed greatly from expectations. We personally traveled to these hospitals to conduct field surveys and obtain data. Before data analysis, we ensured 100% integrity and completeness of the 24 hospitals' data. As the price reforms were enacted on July 1, 2017, we selected the data of the first 3 years before reforms (2014, 2015, and 2016) and the year after reforms (2018) for analysis. This study primarily used principal components analysis to compare among-region differences (30–32).

Principal components analysis was used to measure the price reform performance scores of hospitals. First, we identified six principal components indicators. From the perspective of government evaluation, medical service price compensation and proportion of revenue derived from medicines were selected. From the perspective of hospital operation, the growth rate of hospital medical income and the growth rate of hospitalizations were selected. From the perspective of patient burden, the average outpatient expenses and the average hospitalization expenses were selected. The specific meanings of the six indicators are shown in Table 1. The impact of the price index was considered and excluded from the measurement of the indicator. Second, all indicator data were standardized as z-scores. Finally, principal component analysis was performed on the normalized data. Components with eigenvalues >1 were extracted until the cumulative variance explained exceeded 70%. The score of the principal component was the corresponding principal component analysis multiplied by the arithmetic square root of the corresponding variance. The composite score is obtained by summing the actual scores of all factors. The actual score of the factor is equal to the actor variance percentage multiplied by the initial score. The 24 hospitals were ranked in terms of reform performance via the principal components analysis. Further, regional performance differences were compared.

**Table 1:** Performance evaluation indicators.

**Indicators**	**Meaning**
Medical service price compensation rate	Medical service price compensation income/(pharmaceutical income * actual drug addition rate) * 100%
Medical income growth rate	(Total medical income of a hospital in 2018 − Average medical income in 2014-2016)/Average medical income in 2014-2016 *100%
Decrease in medicine proportion	The proportion of hospital drug revenue in 2018 − the average proportion of drug revenue in 2014-2016
Hospitalization growth rate	(Total number of hospitalizations in 2018 − Average number of hospitalizations in 2014-2016)/Average number of hospitalizations in 2014-2016 * 100%
Per capita outpatient cost growth rate	(2018 per capita outpatient expenses − per capita outpatient expenses in 2014-2016)/per capita outpatient expenses in 2014-2016 * 100%
Per capita hospitalization cost growth rate	(2018 per capita hospitalization expenses − per capita hospitalization expenses in 2014-2016)/per capita hospitalization expenses in 2014-2016 * 100%

## Results

### Price Adjustment Range of Different Service Items in Different Regions

The commonalities among regions were that the prices of medical nursing, treatment, surgery, and Chinese medicine all increased, while the prices of medical examinations decreased. However, there were differences in the price adjustments for the same medical service items in different regions. The key price adjustments for medical service items also differed among regions (Table 2).

**Table 2:** Average price adjustment range of public hospitals in various regions of Guangdong province (%).

**Regions**	**Diagnosis**	**Nursing**	**Bed**	**Treatment**	**Surgery**	**Assay**	**Examination**	**Chinese medicine**
Pearl River Delta	57.45	35.15	15.36	26.70	41.95	−5.50	−4.15	8.32
Eastern region	17.80	37.77	11.31	17.05	27.92	−1.35	−1.87	20.51
Western region	10.29	41.97	11.75	11.00	24.09	−3.10	−2.96	18.56
Northern region	10.62	33.15	−9.29	6.79	19.88	−6.05	−15.22	10.74

### Indicator Analysis

Table 3 shows that the compensation rates in the Pearl River Delta, Eastern, and Northern regions were all above or close to the target of 80%; the Western region did not attain the target. After the price adjustments, the medical and hospitalization-related income of hospitals in all regions increased steadily, and the hospitals operated effectively. In addition, the proportion of income derived from medicines decreased significantly. However, the average cost of outpatient and inpatient admissions continued to show an increasing trend, and growth in some areas was large, such as the average cost of outpatient clinics in the Pearl River Delta region and the Eastern region, and the average cost of hospitalization in the Western region.

**Table 3:** Index values of different regions (%).

**Regions**	**Compensation rate**	**Medical income growth rate**	**Hospitalization growth rate**	**The decreased proportion of drug costs**	**Outpatient cost growth rate**	**Hospitalized patients' cost growth rate**
Pearl River Delta	79.50	10.62	7.99	−4.71	20.12	3.56
Eastern region	84.49	9.06	7.58	−6.15	15.78	4.32
Western region	74.38	15.48	7.49	−4.15	4.26	11.36
Northern region	96.29	15.81	12.28	−6.26	4.38	6.10

### Principal Components Analysis

**Figure 1:**
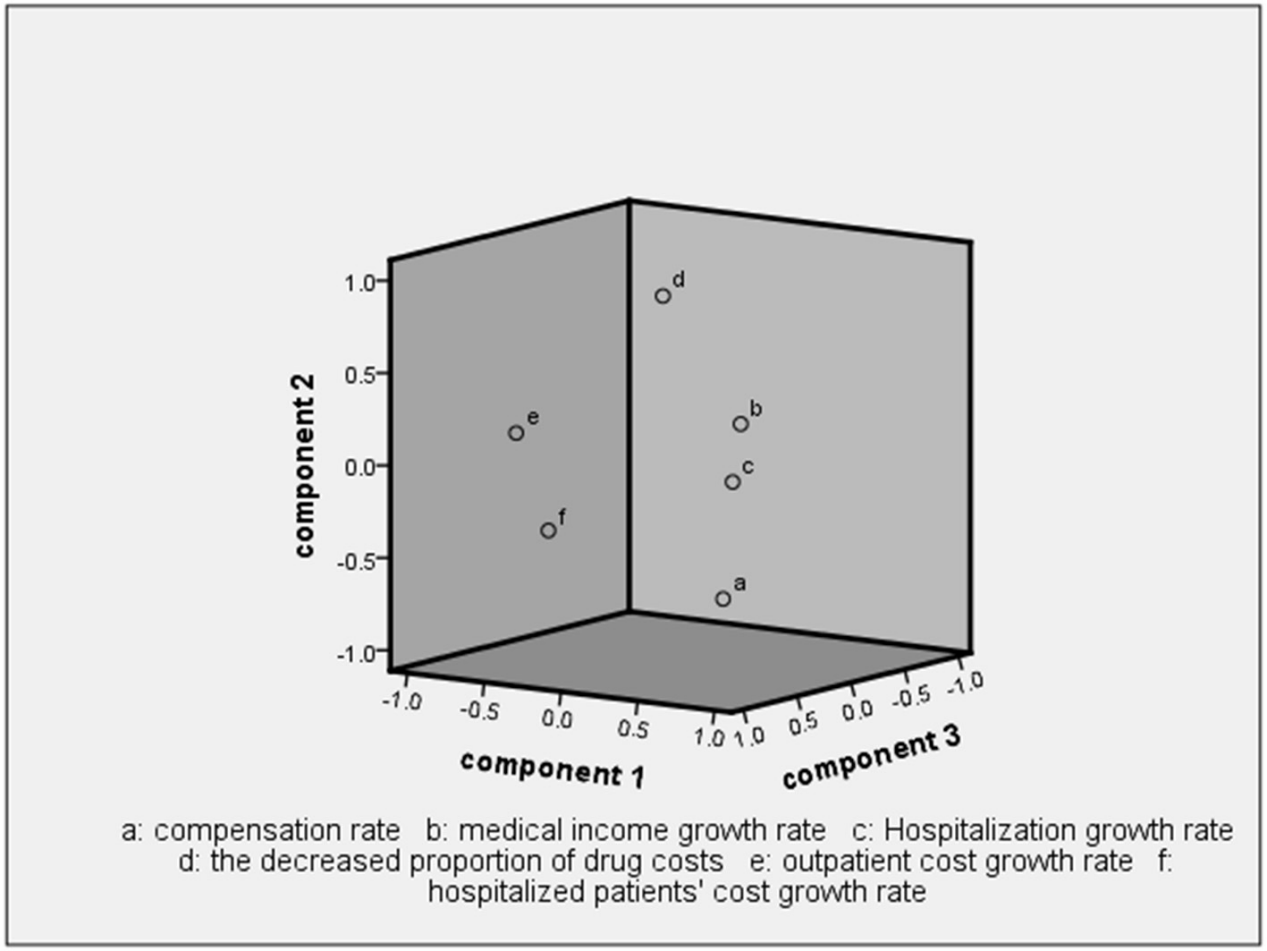
Component plot.

Three principal components were obtained with eigenvalues of λ_1_ = 1.77, λ_2_ = 1.54, and λ_3_ = 1.02, which together explained 71.14% of the underlying variance (Table 4). That is, the three principal components could represent the six initial indicators when analyzing post-reform performance. Therefore, the first three principal components were extracted and are referred to as F1, F2, and F3 hereafter (Figure 1).

**Table 4:** Rotating component matrix and coefficient matrix after conversion.

**Index**	**Rotating component matrix**			**Component score coefficient matrix**		
	***F1***	***F2***	***F3***	***F1***	***F2***	***F3***
Compensation rate	0.12	0.80	−0.11	0.07	0.52	−0.01
Medical income growth rate	0.88	−0.03	0.04	0.60	0.00	0.21
The decreased proportion of drug costs	0.03	−0.86	0.02	0.01	−0.57	−0.04
Hospitalization growth rate	0.58	0.24	−0.02	0.39	0.17	0.12
Outpatient cost growth rate	−0.01	−0.16	0.95	0.18	−0.04	0.86
Hospitalized patients' cost growth rate	−0.68	0.27	0.50	−0.36	0.20	0.35

### Performance Ranking of Hospitals

As shown in Table 5, the performance scores and rankings of the 24 hospitals were obtained by principal components analysis. The performance of comprehensive hospitals was better than that of traditional Chinese medicine hospitals. In addition, the F1 and F3 scores of the general hospitals were higher than F2. This shows that the reforms affected general hospitals predominately in the growth of medical income and inpatient numbers, and the average outpatient and inpatient expenses. The scores for F2 were generally higher than scores for F1 and F3 in traditional Chinese medicine hospitals. This shows that the Chinese medicine hospital has achieved significant results in the compensation rate and the decline in the proportion of medicines.

**Table 5:** Twenty-four hospital performance rankings.

**Hospitals**	**F1**	**F2**	**F3**	**Comprehensive score**	**Performance ranking**
Chinese Medicine Hospital A (Pearl River Delta)	0.00	2.50	1.18	0.87	1
General Hospital A (Pearl River Delta)	2.68	0.26	0.15	0.80	2
General Hospital A (East)	2.26	0.25	0.32	0.72	3
General Hospital B (Pearl River Delta)	0.92	0.71	0.18	0.46	4
General Hospital A (East)	0.03	−0.06	1.32	0.25	5
General Hospital B (East)	−0.98	1.24	0.69	0.19	6
General Hospital B (West)	−0.44	0.19	1.13	0.15	7
Chinese Medicine Hospital A (East)	−0.59	0.87	0.37	0.14	8
General Hospital A (West)	0.12	−0.14	0.49	0.09	9
General Hospital C (North)	1.17	0.86	−2.56	0.03	10
Chinese Medicine Hospital B (West)	−0.09	−0.32	0.68	0.03	11
Chinese Medicine Hospital C (West)	−0.51	0.50	−0.06	−0.02	13
Chinese Medicine Hospital A (West)	0.15	−0.56	0.39	−0.03	14
Chinese Medicine Hospital C (North)	−0.52	−0.32	0.73	−0.08	15
Chinese Medicine Hospital A (North)	0.47	−1.43	0.15	−0.21	16
Chinese Medicine Hospital C (Pearl River Delta)	−0.88	−0.25	0.30	−0.23	17
Chinese Medicine Hospital B (Pearl River Delta)	0.38	−1.18	−0.29	−0.26	18
Chinese Medicine Hospital B (East)	−0.30	0.31	−1.51	−0.29	19
Chinese Medicine Hospital B (North)	−0.30	−0.96	−0.18	−0.36	20
General Hospital B (North)	−0.74	0.94	−2.35	−0.41	21
General Hospital C (West)	−1.38	−0.65	0.33	−0.46	22
General Hospital A (North)	−1.62	0.08	−1.19	−0.63	23
Chinese Medicine Hospital B (East)	0.12	−2.53	−0.51	−0.71	24

### Reform Performance in Different Regions

**Figure 2:**
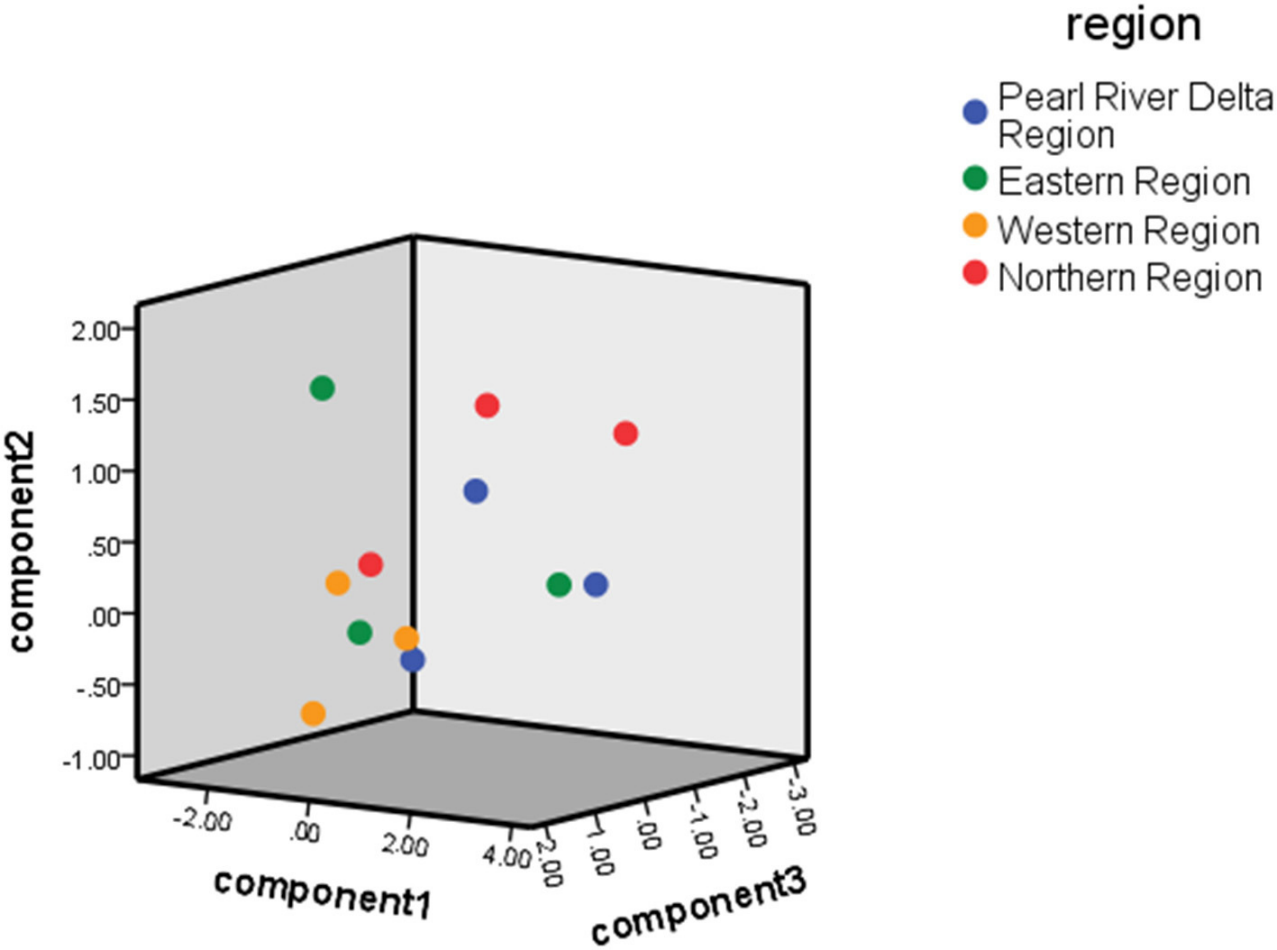
General hospital.

**Figure 3:**
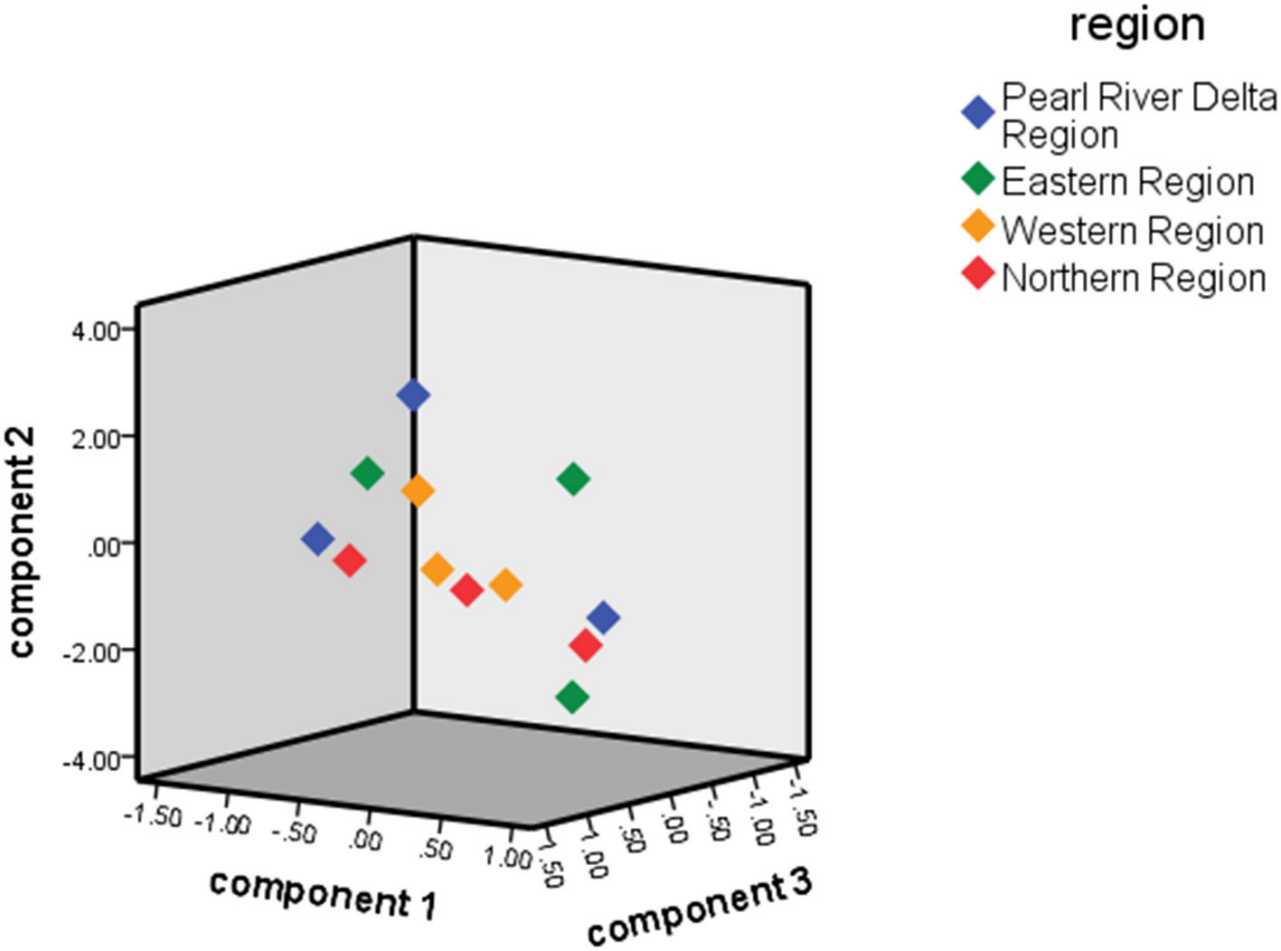
Traditional Chinese medicine hospital.

For comprehensive hospitals, the reform performance scores of the Pearl River Delta region were highest, followed by the Eastern region, Western region, and Northern region. For traditional Chinese medicine hospitals, the reform effect was best in the Pearl River Delta region, followed by the Western region, Northern region, and Eastern region. The ranking of the reform effects for comprehensive hospitals was consistent with the regional economic level. The specific rankings are shown in Table 6. Figure 2 shows the distribution of scores for component 1, component 2, and component 3 of each general hospital. Meanwhile, Figure 3 shows the distribution of scores for the three components of each traditional Chinese medicine hospital.

**Table 6:** Reform performance of various regions in Guangdong province.

**Regions**	**General hospital total score**	**Chinese medicine hospital total score**
Pearl River Delta region	1.24	0.37
Eastern region	1.16	−0.87
Western region	−0.22	−0.02
Northern region	−1.01	−0.65

## Discussion

Based on principal components analysis, we calculated the performance of 24 public hospitals in 12 cities, and compared the overall performance of four regions, focusing on the relationship between regional differences and price reform performance. Further, the effectiveness of the medical service price reform in the province was analyzed, as well as the advantages and disadvantages of the reform. Compared with the existing literature (24–26), the sample selected for this study was sufficiently large, the data more complete and reliable, and the analysis was not restricted to a certain hospital or region. Moreover, this study is the first to explore the specific performance differences related to medical service price adjustment policies in different regions.

This study showed that different service items in different regions exhibited significantly different adjustments in the medical service price. This may be related to differences in economic and basic health status in different regions. The level of regional economic development will affect local public hospitals, including the basic medical equipment available, medical environment, skill of employees, and the amount of attention each patient receives (33). The government needs to consider the status quo of public hospitals when formulating price policies. In addition, when a new price policy is implemented, changes in medical prices will affect the operation and development of public hospitals. A study of Norwegian hospitals found that medical prices fluctuated by 10% and the number of patients increased by 0.8–1.3% (34). Therefore, regional economic development, hospital tapes policy intentions, and medical prices are often in a process of balance and coordination, and tend to be rationalized.

The Pearl River Delta region is located in the south-central part of Guangdong Province and is one of the most developed regions in China (35). The number of public hospitals in the Pearl River Delta region accounts for more than 60% of the total public hospitals in Guangdong Province. The service quality of hospitals is much higher than in the other three regions. The higher economic level and quality of medical care in the Pearl River Delta region has led to generally higher basic fees for public hospitals than in the other three regions. However, its high medical expenses are predominately generated via examinations and consumables, while the cost of technical services such as diagnosis, treatment, and surgery is relatively low. Therefore, the local government generally increased the cost of diagnosis, treatment, nursing, surgery, and so forth, and lowered inspection costs in the Pearl River Delta region during the price reform.

Compared with the Pearl River Delta region, the lower economic levels of the other three regions led to lower hospital fees. These local governments are more concerned regarding how to improve the hospital profitability in order to maintain the normal development of public hospitals. Therefore, local governments have considered not only excessively burdening the people via fees, but also slightly increasing the price of surgery, treatment, and nursing. Among them, the focus has been on raising the price of Chinese medicine services that include government subsidies and preferential policies. At the same time, local governments in the Eastern and Western regions have only slightly lowered the prices of inspections to ensure the normal operation of the hospitals.

The results showed that the performance scores of general hospitals and traditional Chinese medicine hospitals in the Pearl River Delta region were higher than those of the other regions. The Pearl River Delta region is the center of politics, culture, and economy in Guangdong Province (35). Public hospitals in the Pearl River Delta region may be more susceptible to policy. The comprehensive analysis of general hospitals, and the regional performance rankings are consistent with regional economic strength rankings, which indicates that the local economic level is related to the effect of reforms on local general hospitals. Riedel made similar observations, and found that the development of German hospitals faced severe challenges. An important point is that hospital reform and development are constrained by local economic development (36). At present, public general hospitals in China rely primarily on medical income to maintain hospital operations. Therefore, the services provided by the comprehensive hospitals that are most affected by the policy are likely to be severely affected. In addition to Germany, public hospitals in Japan are also generally facing financial deficits. Konosuke confirmed this and suggested that the extent of public hospital development is related to local purchasing power (37). However, prior studies only described the operation of public hospitals in particular locations, and did not engage in in-depth analysis of the differences in policy effects among regions. The current study add such research content. Overall, for regions with different levels of economic development, the same policy may not be applicable, and the effects of policies will be different. Our subsequent studies will assess how to formulate and improve policies for public hospitals in regions with different levels of economic development.

Compared with general hospitals, the reform performance of traditional Chinese medicine hospitals is affected not only by local economics, but also by local government support policies. The more the local government attaches importance to the development of traditional Chinese medicine hospitals, the greater the support it provides.

The development of local traditional Chinese medicine hospitals is better than general hosipitals. However, under the influence of support policies, the medical prices and patient-incurred expenses of traditional Chinese medicine hospitals also increased rapidly. The financial burden on patients in Chinese medicine hospitals has increased. Therefore, the government need to find balance when supporting the development of traditional Chinese medicine hospitals.

Results showed that the policy effect of controlling fees is not straightforward, and the burden on patients continues to increase on a yearly basis. As can be seen from Table 3, the average growth rate of patients' fees is still high, far exceeding the growth rate of the national consumer price index (2.1%) in 2018. The cost of medical treatment for residents has not decreased, and the effect of controlling fees is not satisfactory. The average outpatient expenses in the Pearl River Delta region reached 20.12% of the total medical costs, and the average hospitalization expenses in the Western region reached 11.36% of the total medical costs. These are much higher than expected for the policy developers, adding a considerable burden to patients. Therefore, although the reforms reduced the cost of medicines and inspections, the overall cost of medical care has increased for some patients. Tianlin also found that the cost borne by patients did not significantly decrease after medical service price adjustment in a study of Qingdao City, Shandong Province (7). In addition, Cooper found that hospital medical service prices rose by 42% between 2007 and 2014, and patient-borne costs rose sharply in the US. He suggested that policy makers should consider a range of options to address hospital price increases, including antitrust enforcement, management of pricing, and the use of reference pricing (38). Overall, governments need to develop more effective measures to control the growth of medical expenses.

Principal components analysis showed that the effect of reforms were significantly better on comprehensive hospitals than traditional Chinese medicine hospitals. This may be because the proportion of income derived from inspections and drugs in general hospitals is much larger than that of traditional Chinese medicine hospitals, but the proportion of income derived from technical aspects such as surgery, treatment, and nursing is much lower. After the implementation of the policy, the average cost of patients in general hospitals increased less than that of traditional Chinese medicine hospitals. This also shows that the comprehensive hospitals were greatly affected by the policy, and such hospitals face greater difficulties and challenges than do Chinese medicine hospitals. Similar observations have been made by other researchers. Tang found that the cost of service-dependent hospitals and drug-dependent hospitals was significantly different in a study of public hospitals in Nanjing (8). He proposed that different pricing policies should be developed for different types of hospitals. At present, in China, an identical policy is implemented concurrently across the country or province. Therefore, policies can have different effects in different places and under different conditions. In this case, hospitals are facing increasing pressure, and the contradiction in the requirements of local governments and local medical institutions is prominent.

Tables 2, 3 show that the cost of diagnosis, treatment, surgery, and nursing, which reflect the value of the technical labor of medical professionals, is rising, and inspection costs are reducing. This encourages hospitals to develop technical medical specialties, especially orthopedics and physiotherapy. After the reform, more hospitals are willing to carry out medical surgery that requires advanced technology because they now have sufficient income. The policy has somewhat reduced the occurrence of over-examination in hospitals, because the profit associated with inspections has been reduced. From this perspective, the change to hospitals' income structures was benign, and the value of the labor of medical professionals has been recognized and valued. Li proposed that county-level public hospitals tend to have a reasonable income structure after adjusting for medical services (24). Wei suggested that the significance of this price adjustment is to make the price of medical services reflect the labor value of medical staff (5). Moreover, both researchers believed that the policy would have a positive impact on the internal management and performance of hospitals. This is consistent with the results of the current study.

There are also several limitations to this study. First, although principal components analysis was suitable for comparing the effects of reforms among hospitals or regions, the effects of reforms at the individual hospital level was not studied in depth. In future, it is necessary to further study the specific impact of the price adjustment policy within individual hospitals. Second, this study did not assess the different responses of local governments in implementing policies. Third, this we proposed that regional differences in economic development will influence the effects of policy changes, but the specific effects have not been thoroughly studied. These aspects represent important future research directions of our research team.

## Conclusions

At present, the reform of medical service prices has achieved some acceptable results in public hospitals in Guangdong Province, but there remain issues. According to the analysis reported here, the following questions and suggestions are proposed: First, the current government's pricing policy does not distinguish between hospital types, such as general hospitals, traditional Chinese medicine hospitals, and specialized hospitals, which is unfair to hospitals. Different types of hospitals carry out different services, so the effects of policies often vary widely. Therefore, formulating appropriate policies is a powerful measure by which to resolve current contradictions regarding different types of hospitals in regions with different levels of economic development. Second, the price adjustment of medical services improved the internal income structure of hospitals, but did not reduce patients' expenses. Therefore, the government's subsequent policy development could usefully focus on the control of patient-borne costs. Third, the current price of medical services varies among cities, which will lead to the flow of patients between cities, and put pressure on local medical institutions. Therefore, we recommend implementing a given price policy among cities with similar economic and healthcare levels, eventually forming a regional medical service pricing mechanism. Fourth, few studies have applied advanced statistical methods to the field of medical service pricing. Therefore, we recommend using such methods within this field.

## Data Availability Statement

The original contributions presented in the study are included in the article/supplementary material, further inquiries can be directed to the corresponding author/s.

## Author Contributions

W-QL participated in research and report preparation. Y-BW is responsible for full text guidance. All authors contributed to the article and approved the submitted version.

## Conflict of Interest

The authors declare that the research was conducted in the absence of any commercial or financial relationships that could be construed as a potential conflict of interest.
